# Dental Caries in Children and Its Relationship with Parenting Styles: A Systematic Review

**DOI:** 10.3390/children11111324

**Published:** 2024-10-30

**Authors:** María Moya-López, Ana Ruiz-Guillén, Martin Romero-Maroto, María Carrillo-Díaz

**Affiliations:** 1 Doctoral Program in Health Sciences, International PhD School, Rey Juan Carlos University, 28922 Alcorcón, Madrid, Spain; maria.moya@urjc.es; 2 Orthodontic and Pediatric dentistry Department, Rey Juan Carlos University, 28922 Alcorcón, Madrid, Spain; martin.romero@urjc.es (M.R.-M.); maria.carrillo@urjc.es (M.C.-D.)

**Keywords:** parental styles, caries, carious, child, infant

## Abstract

Background: It can be affirmed that the parenting style of parents has an impact on the health-related behaviors of their children; the environment that parents create for their children can have an impact on both their habits and their oral health, and on the incidence of dental caries in children. The purpose of this study was to analyze the association between parenting style and childhood dental caries. Methods: Two researchers independently searched the English literature published up to May 2024 in four databases (PubMed, Web of Science, Scopus y Cochrane Library). The risk of bias was evaluated using the Modified Newcastle–Ottawa Quality Assessment Scale (NOS). This study is registered on PROSPERO (CRD42024573447). Results: Of the 130 studies identified, nine of them, with a total of 4250 participants, met our inclusion criteria. The evidence on the relationship between parenting styles and dental caries is varied. Of the studies reviewed, three showed a significant association between both factors, while four found no correlation, and two reported no significant differences in relation to parenting styles and the occurrence of dental caries. Conclusions: This discrepancy emphasizes the need for further research. Parenting styles impact child dental behavior on a global level, highlighting the relevance of recognizing these approaches in a dental context, given that parents have a fundamental role in guiding their children’s behaviors.

## 1. Introduction

Dental caries is a multifactorial infectious disease associated with an imbalance of the oral microbiome, known as dysbiosis, and the elevation of cariogenic bacteria. This process leads to the demineralization of the hard tissues of the tooth, such as enamel and dentin, due to acids generated by the bacteria-metabolizing sugars present in the diet [1].

Caries is the result of the interaction of multiple factors. These include socioeconomic factors (social class, occupation, income, and education level), demographic characteristics (age, sex, and ethnicity), behavioral factors (non-use of fluoride dentifrice, sugar consumption, poor oral hygiene, and lack of preventive dental care), and biological factors (recent caries experience/active caries lesions, biofilm retentive factors, developmental defects of the enamel, disabilities, saliva amount and quality, cariogenic biofilm) [2].

This condition can significantly affect the physical, mental and social well-being of children [3], so it is essential to investigate its origin for effective prevention and treatment. Although progress has been made in understanding the etiology of caries, ranging from biological to social and psychological factors [4], challenges related to parenting styles persist, which justifies continued research in this field.

Childhood dental caries is one of the most prevalent diseases globally, with an estimated prevalence of 46.2% in primary teeth [5]. Although caries rates have declined in high- and middle-income countries in recent decades, it remains a major public health problem in both developed and developing countries, despite being largely preventable [6]. Children’s oral health and oral health habits are largely determined by their primary caregivers [4]. Parental practices, such as feeding styles or supervised toothbrushing with fluoride toothpaste, as well as general parenting styles, may be linked to the occurrence of caries [7,8,9].

Specifically, parenting style is defined as the emotional environment in which parent–child interactions take place. This is measured on the basis of the “responsiveness” and “demandingness” that parents exhibit toward their children, with responsiveness being understood as the degree to which parents show warmth, acceptance, and involvement, and demandingness referring to the degree to which parents exhibit control and supervision in the upbringing of their children [10]. Four parenting styles stand out: authoritarian, authoritative, permissive [11], and neglectful [10]. Authoritarian parents represent high control and low warmth, authoritative parents show high warmth and high control, permissive parents promote high warmth and exhibit low behavioral control, and neglectful, or uninvolved, parents have lax behavioral control and provide minimal emotional support to their child [12]. There is little research on the latter style, as few parents fall into this category and, moreover, are unlikely to volunteer for research studies [13].

It is widely accepted [4,14,15] that parents’ beliefs about their ability (self-efficacy) to manage oral health and related habits play a crucial role in shaping the preventive behaviors that will reduce the occurrence of dental caries in their children. Research indicates that parents with authoritarian and permissive parenting styles tend to be less influential in promoting proper oral hygiene practices in their children [16,17]. A connection has been demonstrated between authoritative parenting styles and positive behaviors in children, such as the adoption of healthier eating habits and a reduction in the consumption of sugar-sweetened beverages [18,19].

However, little attention has been paid to how overall family function and relationships influence children’s oral health. For researchers in this field, it is essential to use reliable, valid, and appropriate assessment tools to study the connection between family relationships and children’s oral health [20].

Evidence supports a possible relationship between parenting styles and dental caries [21], but research is limited on the topic; this is due to the limited number of studies, combined with variations in the study objectives, methodology, and outcome variables, all of which have limited the ability of researchers to draw clear conclusions about the nature and strength of the associations between parenting style and dental caries in children. Therefore, the present systematic review aimed to analyze the association between parenting style and childhood dental caries in previous studies.

## 2. Materials and Methods

### 2.1. Review Design

This review was performed according to the Preferred Reporting Items for Systematic Review and Meta-Analysis (PRISMA) guidelines. The protocol was registered in the International Prospective Register of Systematic Reviews (PROSPERO; registration number: CRD42024573447). Articles published until April 2024 were included in this review. The research question was as follows: How do parenting styles influence the incidence of caries in children? The checklist of PRISMA parameters is presented in the Supplementary Material (Supplement S1).

### 2.2. Literature Search and Bibliographical Sources

Two investigators independently searched four electronic databases (PubMed, Web of Science, Scopus y Cochrane), using the keywords described below: (parental styles) AND (caries) OR (carious) AND (child) OR (infant). The final search, including that of articles published electronically before being printed, was conducted on 3 June 2024. A manual search was performed using the reference lists of previous reviews. The detailed search strategy is presented in the Supplementary Material (Supplement S2). The reference lists of the included studies and the related studies that were not included were reviewed in an attempt to identify additional studies.

### 2.3. Study Selection

After eliminating duplicates, two investigators independently screened the titles and abstracts of the retrieved articles according to the inclusion criteria [20] listed in Table 1. To avoid excluding potentially relevant articles, abstracts with unclear results were included in the full-text analysis. Any disagreement was resolved via discussion. In the case of disagreement, a third investigator was consulted for the final decision. Eligible articles were selected based on an evaluation of the full text of all remaining studies according to the inclusion and exclusion criteria.

Studies on children outside the age range of 3–7 years; studies in which parents did not participate; and studies focusing on examining a physical or mental illness, a behavioral problem of the parent or child that could affect oral health, or general parenting styles were excluded. Studies related to feeding style rather than parenting style or feeding behaviors, or those that did not measure the association between parenting and childhood dental caries, were excluded [22]. Literature reviews, case reports, case series, letters, commentaries, and protocols were excluded.

### 2.4. Data Extraction and Analysis

Two reviewers independently assessed the methodological quality of the studies and checked the tools used to measure parenting style and dental caries for potential sources of bias, attrition, the validity of the survey instruments, and reliability within their respective study populations [23].

The following extracted data considered relevant to this review were incorporated into a predesigned data collection form in Microsoft Excel (microsoft 365): study identification data (first author’s name, year of publication), study design and dental setting, participant data (sample size and mean age), parenting style assessment criteria, dental caries assessment criteria, other variables assessed, and results. Discrepancies were resolved via discussion.

### 2.5. Risk of Bias

The risk of bias of the included studies was assessed independently by the same two investigators by using a modified version of the Newcastle–Ottawa Scale (NOS) quality assessment tool [24]. The included observational studies were assessed primarily on eight methodological elements. Any disagreement between the two reviewers was resolved through discussion with a third investigator.

## 3. Results

### 3.1. Study Selection

**Figure 1:**
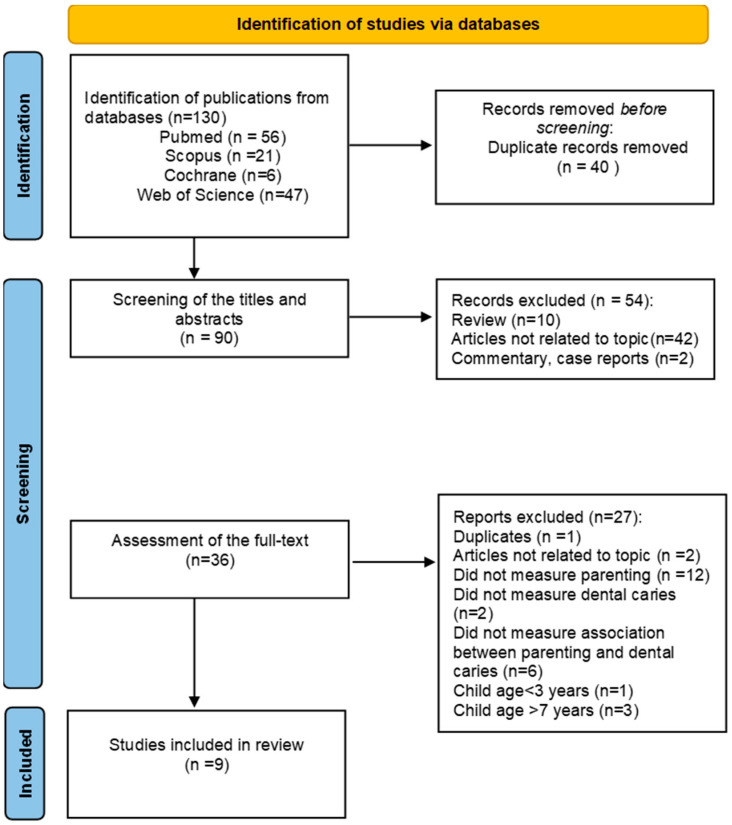
PRISMA flow diagram [23].

A total of 130 publications from four databases were identified (56 from PubMed, 21 from Scopus, 6 from the Cochrane Library and 47 from Web of Science). Forty duplicates were eliminated. After screening the titles and abstracts, 54 publications describing clinical cases, commentaries, reviews, or off-topic articles were excluded. The full texts of the remaining 36 articles were retrieved, and 27 were excluded either because the studies included participants with a mean age of less than 3 years (n = 1) and a mean age of more than 7 years (n = 3), were on another topic (n = 2), did not investigate parenting styles (n = 12), did not investigate dental caries (n = 2), or did not investigate the relationship between parenting styles and dental caries (n = 6). There was one duplicate study (n = 1). Finally, nine articles were selected for data extraction. No additional publications were found in the reference lists of the included studies. The two investigators had 97% agreement on the independently selected articles. The nine articles were also independently assessed for bias and study quality criteria. A flow diagram of the literature search process is presented in Figure 1.

### 3.2. Study Characteristics: Study Design and Population

Table 2 provides a general description of the included studies.

The studies were carried out in six countries, including the USA, Singapore, Saudi Arabia, Korea, Sri Lanka and India, with the latter accounting for four studies. The scientific output in the area was the highest in 2020 (33%), followed by 2023 (22%). Except for one case–control study, all studies were considered cross-sectional observational studies.

In total, 44% of the articles focused on children from 3 to 6 years old and 22% focused on children from 3 to 5 years old.

In all studies, dental data were obtained by performing intraoral examinations. The presence of caries was recorded, using the dmft index. However, only four articles indicated whether caries was present or not, with a dichotomous response (positive/negative).

Regarding the parenting style, all of the studies used self-reported questionnaires administered to parents. Each study used only one tool to measure parenting style. In total, 89% of the studies used the Parenting Style and Dimensions Questionnaire (PSDQ), which was designed to assess Baumrind’s [25] typology for three dimensions of parenting (authority, authoritarianism, and permissiveness).

**Table 2:** Characteristics of the included studies.

Authors	Population/ Patients	Parenting Style	Caries Assessment	dmft Scores (Mean, SD)	Dental Caries (N/%)	Results
Howenstein et al. (2015) [16]	132 children (3–6 years old)	PSDQ (parents)	‘positive’, or ‘negative’	NR	Authoritative: 17 (20%) Authoritarian: 10 (91%) Permissive: 32 (97%) Total: 59 (45%)	Children with authoritative parents exhibited less caries compared to children with authoritarian and permissive parents.
Viswanath et al. (2020) [26]	315 children (3–7 years)	PSDQ (parents)	DMFT/deft index	NR	Authoritative: 106 (44%) Authoritarian: 16 (53%) Permissive: 39 (87%) Total: 161 (51.1%)	Children of both authoritative and authoritarian parents had low caries status and the permissive parenting style showed a threefold increase in caries status when compared to the authoritative parenting style.
Shalini et al. (2023) [27]	1216 children of 3–6 years old preschool	PSDQ (parents)	‘positive’, or ‘negative’	NR	Authoritative: 355 (41.6%) Authoritarian: 137 (55.2%) Permissive: 55 (47.8%) Total: 547 (45%)	Children subjected to the authoritarian parenting style were more prone to caries. Children subjected to the authoritative parenting style were more caries-free.
Quek (2021) [28]	389 children of 4–6 years	PSDQ (parents)	dmft index	5.4 ± 5.0	Total: 285 (73.3%)	Parenting styles were not associated with dmft.
Alagla (2019) [29]	280 children of 3–6 years old	PSDQ (parents)	dmftindex	NR	Authoritative: 254 (95.8%) Authoritarian: NR Permissive: 17 (100%) Total: 271 (96%)	The correlation between parenting style and dental caries among children was not significant.
Dabawala et al. (2017) [30]	422 children of 3–5 years	PSDQ (parents)	‘positive’, or ‘negative’	NR	Father Authoritative: 206 (50.2%) Authoritarian: 1 (50%) Permissive: 4 (40%) Mother Authoritative: 205 (49.6%) Authoritarian: 4 (66.6%) Permissive: 2 (66.6%)	The difference in the parenting style of parents of children with and without ECC based on the father and mother’s self-report of their own and spouse’s parenting style was not significant.
Miso Lee et al. (2019) [31]	158 children of 3–6 years	PSDQ (parents)	dft index	Authoritative: 2.77 (N = 151) Authoritarian: 5 (N = 6) Permissive: 7 (N = 1) Total: 3.6	NR	The difference between the dft index, according to the three groups, was not statistically significant.
Sabbarwal et al. (2020) [9]	300 children of 3–5 years	PSDQ (parents)	dmft index	Authoritative: 1.51 ± 1.94 Authoritarian: 2.1 ± 1.67 Permissive: 4.25 ± 3.43 Total: 2.28 ± 2.66	Authoritative: 88 (45.8%) Authoritarian: 27 (90%) Permissive: 68 (87.1%) Total: 183 (61.0%)	The permissive group had a higher mean dmft.
Gunasinghe et al. (2023) [32]	1038 children of 3–4 years	Local measure (Udayamalee, 2013) (mothers)	‘positive’, or ‘negative’	NR	Total: 584 (56.3%)	Parenting style was a relevant secondary factor influencing children’s oral health habits, such as the consumption of sweets, dental hygiene and dental visits, contributing to the development of early childhood caries.

Abbreviations: PSDQ, parenting styles and dimensions questionnaire; NR: not report.

Measures of other variables, such as child anxiety scales, eating habits, or oral hygiene measures, among others, were included in all studies, but were not interpreted in the present study.

After analyzing the results of the articles, it was determined that dental caries was significantly associated with parenting style in three studies; four studies found no association between parenting style and caries; and two found no significant differences between parenting styles and caries.

### 3.3. Risk of Bias

The risk of bias in the included articles was assessed according to the Newcastle–Ottawa scale and is presented as a quality score in Table 3. Except for one study [27], all of the studies were rated as high quality.

## 4. Discussion

Five of the studies found an association between parenting styles and dental caries [9,16,26,27,32]; however, in two of these articles, the relationship was not significant [27,32]. The remaining four studies found [28,29,30,31] no correlation between dental caries and parenting style. In the study by Alagla et al. [29], the non-association could be related to the fact that oral-hygiene-related dental caries is a multifactorial process, such that parenting is one factor among other distinct ones. The other explanation could be that children had increased dental caries due to the high consumption of a cariogenic diet, the lifestyle of the Saudi population, and a lack of effective tooth brushing. In the studies by Lee et al. [31] and Dabawala et al. [30], the association between parenting style and ECC was not determined, as the three types of parenting styles could not be differentiated in the sample. The skewed distribution of parenting styles in these studies may explain the lack of correlation between parenting styles and caries (caries status, dmft); however, another important reason may be that caries is a multifactorial chronic disease and that there are other risk factors that may play a role, in addition to the impact associated with parenting styles [28].

There was a higher percentage of authoritative parents in the studies compared to other parenting styles [9,16,26,27,28,29,30,31]. The results of some of these studies [16,27,31] show that children of authoritative parents have a lower caries rate compared to those of permissive and authoritarian parents, indicating that parental behavior may influence the development of children’s oral habits [33]. Specifically, the study by Howenstein et al. [16] found that authoritative parenting was associated with more desirable child behavior and less dental caries compared with the other two parenting styles. Authoritative parents set rules clearly and use reasoning to enforce them [26]. The above finding could be attributed to the fact that authoritative parents have better control over their children’s diet and restrict children’s access to cariogenic diets. Authoritative parents are more likely to follow up on their children’s dental checkups and implement better oral hygiene practices [29].

The studies by Howenstein et al. [16] and Shalini et al. [27] indicated an association between authoritarian parenting and increased caries, contrasting with that of Viswanath et al. [26], who found that children of authoritarian parents had a low rate of caries. The increase in caries in this group seems to contradict what would be expected from authoritarian households characterized by strict rules. If there were strict rules for children to brush their teeth and follow a specific diet, they would comply. These results could indicate that oral health is not a priority in these families. Another possible explanation is that oral hygiene practices and dietary measures are not reinforced due to low parental responsiveness in these households [27].

In addition, the number of authoritarian households was limited in the studies compared to authoritative and permissive households, so a larger sample size in future research may help to elucidate these findings.

Permissive parenting was associated with increased caries [9,16,26]. This increase in caries in permissive households could be due to the fact that children are free to consume cariogenic foods without restrictions and can decide whether to brush their teeth or not, without facing discipline from their parents. These parents are characterized as lenient, allowing children to set their own rules and act as co-owners of the home. These children are often very demanding and throw tantrums when their wishes are not met. Because permissive parents avoid confrontation and tend to spoil their children, children may choose to misbehave, receiving comfort instead of discipline [16,26].

Regarding the limitations found, the use of a cross-sectional design in all studies may limit the investigation of the role of parenting style, since this type of design only offers a view of a specific moment in time, without capturing changes or trends over time. However, parenting style has long-term effects on child behavior [30]. This limitation prevents definitive conclusions regarding whether parenting style affects the development of caries in children throughout their growth. Conducting longitudinal cohort studies may reveal more about the temporal relationship between parenting style and ECC.

Another limitation is the possible selection bias, given that most of the participants were authoritative parents. However, this could be a representative distribution in today’s society. The uneven distribution of the sample among different types of parenting may restrict our understanding of the relationship between parental behavior and child oral health. Therefore, it is recommended that random sampling techniques be used to ensure that parenting patterns are adequately reflected in the research [9].

It is also important to take into account information bias, given that the data collection was based on parental reports, which can be subject to memory errors or the misinterpretation of the questions. In addition, confounding is a limiting factor, as variables not considered, such as diet or genetics, could influence the observed relationship between parenting styles and dental caries.

Reporting on the parenting practices of both parents would allow the direct comparison and assessment of differences in how each parent impacts the oral health of their children [34].

The findings of this review have important implications for dental practice and parental education. To improve dental health in children, it is recommended that educational programs that promote authoritative parenting styles are developed.

## 5. Conclusions

In conclusion, these results highlight that parenting styles influence child dental behavior globally and underscore the importance of identifying these styles in a dental setting, as parents play a crucial role in guiding their children’s behavior. However, more research is needed to explore the complex nature of parental attitudes, the consistency of oral health behavior, and their role in the development of caries.

## Figures and Tables

**Table 1:** Inclusion criteria.

Studies	Case–Control, Cross-Sectional and Observational Studies
Participants	Children aged between 3 and 7 years, with or without dental caries
Intervention	Parental styles
Outcome measures	Reported outcome measures of both parenting style and oral health; measured association between parenting style and childhood dental caries

**Table 3:** Quality of the studies on the Modified Newcastle–Ottawa quality assessment scale.

Quality of the Studies									
Author (Year)	Selection				Confounding Factor	Outcome			Total
	1	2	3	4	5	6	7	8	
Howenstein et al. (2015) [16]	0	1	1	1	1	1	1	1	7
Viswanath et al. (2020) [26]	1	1	1	0	1	1	1	1	7
Shalini et al. (2023) [27]	1	1	1	0	0	1	1	0	5
Quek et al. (2021) [28]	1	1	1	1	1	1	1	0	7
Miso Lee et al. (2019) [31]	0	1	1	1	1	1	1	0	6
Sabbarwal et al. (2020) [9]	1	1	1	0	1	1	1	0	6
Gunasinghe et al. (2023) [32]	1	1	0	1	1	1	1	0	6
Alagla et al. (2019) [29]	1	1	1	0	1	1	1	0	6
Dabawala et al. (2017) [30]	1	1	1	1	1	1	1	1	8

## Data Availability

Not applicable.

## Supplementary Materials

The following supporting information can be downloaded at: https://www.mdpi.com/article/10.3390/children11111324/s1. Supplement S1: The checklist of PRISMA parameters. Supplement S2: Details of the search strategy.
