# Supplementary material for: Dental Caries in Children and Its Relationship with Parenting Styles: A Systematic Review
Source: Children (Basel). 2024 Oct 30;11(11):1324. doi: 10.3390/children11111324 (PMC11592684; doi:10.3390/children11111324)
Supplement: Supplementary file 1 [file children-11-01324-s001.zip › Supplement S2. The search strategy.pdf]

Search strategy:

((parental styles) AND ((caries) OR (carious))) AND ((child) OR (infant))

Total: 130 items

PUBMED: (56)

((parental styles) AND ((caries) OR (carious))) AND ((child) OR (infant))

WoS: (47)

((TS=(parental styles)) AND TS=(caries OR carious)) AND TS=(child OR infant)

Scopus: 21

(TITLE-ABS-KEY (parental AND styles) AND TITLE-ABS-KEY (caries) OR TITLE-ABS-KEY (carious) AND TITLE-ABS-KEY (child) OR TITLE-ABS-KEY (infant))

Cochrane: 6

ID	Search
#1	parental styles
#2	caries
#3	carious
#4	child
#5	infant
#6	#1 and (#2 or #3) and (#4 or #5)
